# Case Report: Myeloid neoplasms with the t(3;12)(q26.2;p13.1)/*MECOM-ETV6* translocation: report of two new cases and review of the literature

**DOI:** 10.3389/fonc.2025.1526044

**Published:** 2025-04-04

**Authors:** Soumya Mikkilineni, Juan Pablo Pineda-Reyes, Lindsay Wilde, Andres Ferber, Zi-Xuan Wang, Stephen Peiper, Guldeep Uppal, Jerald Gong, Jinglan Liu

**Affiliations:** ^1^ Hematopathology, Department of Pathology and Genomic Medicine, Thomas Jefferson University, Philadelphia, PA, United States; ^2^ Department of Pathology and Genomic Medicine, Thomas Jefferson University, Philadelphia, PA, United States; ^3^ Division of Hematologic Malignancy and Stem Cell Transplantation, Department of Medical Oncology, Sidney Kimmel Cancer Center, Thomas Jefferson University, Philadelphia, PA, United States; ^4^ Department of Medical Oncology, Sidney Kimmel Cancer Center, Thomas Jefferson University Torresdale Hospital, Philadelphia, PA, United States; ^5^ Molecular and Genomic Pathology Laboratory, Division of Genomic Pathology, Department of Pathology and Genomic Medicine, Thomas Jefferson University Hospital, Philadelphia, PA, United States; ^6^ Clinical Cytogenomics Laboratory, Division of Genomic Pathology, Department of Pathology and Genomic Medicine, Thomas Jefferson University Hospital, Philadelphia, PA, United States

**Keywords:** t(3;12), *MECOM*, *ETV6*, cytogenetics, myeloid, neoplasms, *AF4*

## Abstract

The *MECOM* (*MDS1* and *EVI1* complex locus) gene, located at 3q26.2, encodes an oncogenic transcription factor implicated in multiple signaling pathways. Rearrangements involving *MECOM*/3q26.2, including inversions, translocations, insertions and cryptic chromosomal changes, are observed in myeloid neoplasms and are associated with high-risk disease features and poor clinical outcomes. The translocation t(3;12)(q26.2;p13.1) is a rare genetic event, resulting in a fusion of the *MECOM* gene at 3q26.2 with the *ETV6* gene at 12p13.1. To date, only 78 cases of hematologic neoplasms harboring t(3;12) have been reported in the English literature, primarily as case reports or case series. T(3;12) has been associated with abnormalities of chromosome 7, multiple hematopoietic lineage dysplasia, and poor prognosis. Given its rarity, studies on t(3;12) in myeloid neoplasms are limited. In this report, we present two additional cases exhibiting t(3;12), initially identified through routine karyotyping. The clinicopathological, cytogenetic and molecular genetic characteristics were summarized and discussed. A comprehensive review of partner genomic loci and genes mutated in myeloid neoplasms with *MECOM* rearrangement was conducted. The *AF4* gene and the transcription elongation control pathways are proposed as potential therapeutic targets for *MECOM*-rearranged myeloid neoplasms.

## 1 Introduction

Leukemia is a heterogeneous disease characterized by distinctive biological and clinical manifestations, driven by a variety of acquired genetic alterations. Chromosomal translocations involving the *MECOM* locus at 3q26.2 account for less than 1% of myeloid neoplasms. These translocations can be found in both primary and therapy-related cases and are associated with unfavorable clinical outcomes (1–3).

*MECOM* encodes a transcription factor, the mis-expression of which triggers leukemogenic effects through the dysregulation of its direct target genes (e.g., *GATA2, PBX1, PML*, etc.). Further downstream events play critical roles in upregulating protein 1, counteracting the growth-inhibitory effects of TGF-β, co-activating RAS/receptor tyrosine kinase pathway, and inhibiting differentiation and apoptosis. However, the precise functions and underlying molecular pathological mechanism by which MECOM induces leukemogenesis remain poorly understood (2, 4).

Myeloid neoplasms involving *MECOM* rearrangements can be classified into two groups. The first group is the classic type, which consists of inv (3)(q21q26.2) or t(3;3)(q21;q26.2), characterized by an apparent *MECOM*::*RPN1* fusion resulting from a reciprocal chromosomal alteration. The second group is the non-classic type, represented by apparent fusions between the *MECOM* locus and genes other than *RPN1*. The involved chromosomal alterations in this group can be either balanced or unbalanced (2). The t(3;12)(q26.2;p13.1) resulting in an *MECOM::ETV6* (formally named *TEL*) fusion, a non-classic type, is found in less than 0.1% of myeloid neoplasms (4). To date, only 78 cases have been reported in literature, and their clinical and molecular features have been reviewed and summarized by Ronaghy A. et al. (2021) (4). Herein, we describe the clinicopathological, cytogenetic, and molecular genetic features of two new cases. A comprehensive review of partner genomic loci and genes mutated in myeloid neoplasms with *MECOM* rearrangement was also conducted. Online data mining and pathway analysis have highlighted a possible pivotal role of the AF4 transcription elongation factor in these cases.

## 2 Clinicopathologic characteristics

**Figure 1:**
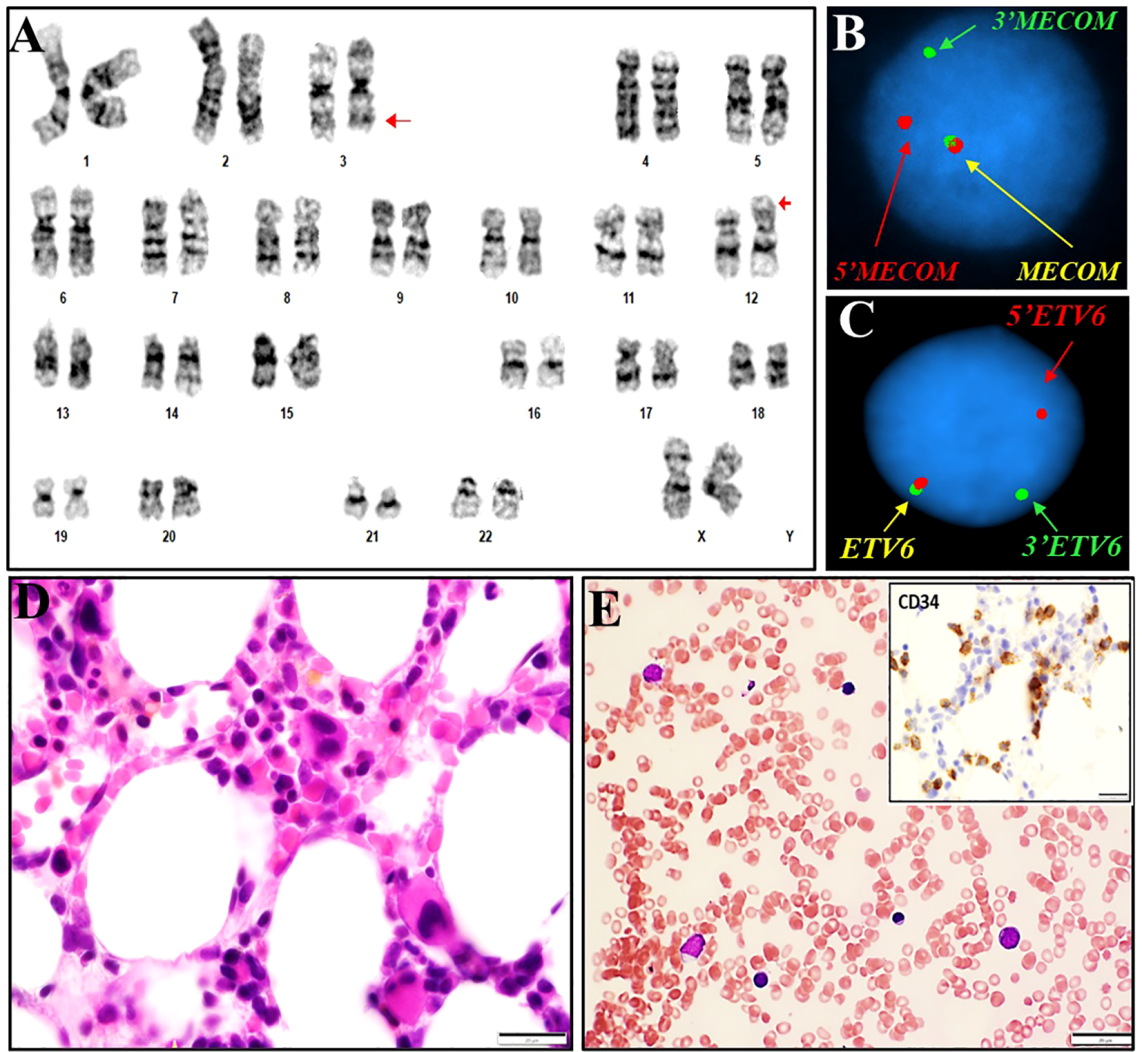
Cytogenetics, FISH and histomorphological findings of case #1. **(A)** Representative karyogram of a translocation 46,XX,t(3;12)(q26.2;p13) observed in 20/20 metaphases. Arrows indicate aberrant chromosomes. **(B)** FISH using dual-color break-apart probes for *MECOM* 3q26.2 demonstrating one normal fusion signal, one split signal for 5’ end region of *MECOM* (green), and one split signal for the 3’ end region of *MECOM* (red), consistent with rearrangement at *MECOM* locus. **(C)** FISH using dual-color break-apart probes for *ETV6* at 12p13.1 demonstrating one normal fusion signal, one split signal for 5’ end region of *ETV6* (red), and one split signal for the 3’ end region of *ETV6* (green), consistent with rearrangement at *ETV6* locus. **(D, E)**: Bone marrow biopsy and touch prep showing dysplasia in erythroid precursors.

Case #1 is a 55-year-old female with a past medical history of early-stage melanoma, 17 years post-excision, who presented to our institution with easy bruising and fatigue. A complete blood count (CBC) revealed pancytopenia. Bone marrow biopsy showed hypocellular marrow (10-20% cellularity) with myeloid immaturity and increased CD34 and CD117-positive myeloblasts (15%). Multi-lineage dysplasia was suggested by the presence of decreased granulocytes with pseudo-Pelger-Huët anomaly (PPHA), megaloblastic erythrocytes with anisocytosis, poikilocytosis, and schistocytes, as well as immature monocytes (**Table 1**, **Figures 1D, E**). A diagnosis of myelodysplastic syndrome with excess blasts-2 (MDS-EB2) was made. The patient was initially treated with decitabine. Unfortunately, after one cycle of decitabine, a bone marrow examination revealed disease progression to hypocellular acute myeloid leukemia with 50-60% blasts. An aggressive induction approach with FLAG-Ida-venetoclax was then administered, and complete remission (CR) and negative minimal residual disease (MRD) status were achieved two months later. After completing one cycle of FLAG-Ven (without idarubicin) for consolidation, she underwent a haploidentical allogeneic hematopoietic stem cell transplant (HSCT). At the latest follow up, the patient was 834 days post-allogeneic HSCT, doing exceptionally well, and with return to baseline functional status and resolution of all symptoms. The most recent CBC reveals only mild anemia with no other concerning abnormalities. Engraftment studies have consistently shown >99% donor cells (**Table 1**). This patient was lost to follow-up due to a family relocation.

**Table 1:** Clinicopathologic, cytogenetic and molecular genetic features of two new cases with t(3;12)(q26.2;p13.

	Case 1	Case 2
**Age at Diagnosis**	55	74
**Sex**	F	M
**Diagnosis**	MDS with EB-2, with hypocellular AML transformation/progression	Myeloid neoplasm with ringed sideroblasts
**Bone marrow blasts (%)**	15% at diagnosis 50% at AML transformation	5-7%
**WBC count (×10^3^/μl)**	3.0	8.3
**Hemoglobin (g/dl)**	8.7	9.4
**Platelets (×10^3^/μl)**	66.0	347.0
**Dysplastic granulocytes**	Pseudo Pelger-Huet anomaly	No
**Dysplastic erythrocytes**	Macrocytosis, Anisocytosis, Poikilocytosis, rare schistocyte	Dysplastic erythroid hyperplasia
**Dysplastic megakaryocytes**	No	Increased megakaryocytic proliferation with dysplasia
**Chemotherapy**	FLAG-Ida-Ven, FLAG-Ven, completed	Azacitidine, incomplete
**HSCT**	Yes, allogeneic	No
**CR**	Yes	No
**OS**	834 days s/p HSCT remains in CR	Deceased 8 months later
**Gene Mutations**	*EZH2, SF3B1*	*ASXL1, DNMT3A, JAK2, SETBP1*, *SF3B1*
***FLT3*-ITD**	Negative	Negative
***MECOM* rearrangement**	Positive	Positive
***ETV6* rearrangement**	Positive	Positive
**Karyogram**	46,XX,t(3;12)(q26.2;p13.1)[20]	46,XY,t(3;12)(q26.2;p13.1),del(7)(q11.2q32)[10] /46,XY[10]

CR, complete remission; *FLT3*-ITD, *FLT3* internal tandem duplication; HSCT, hematopoietic stem cell transplant; OS, overall survival.

**Figure 2:**
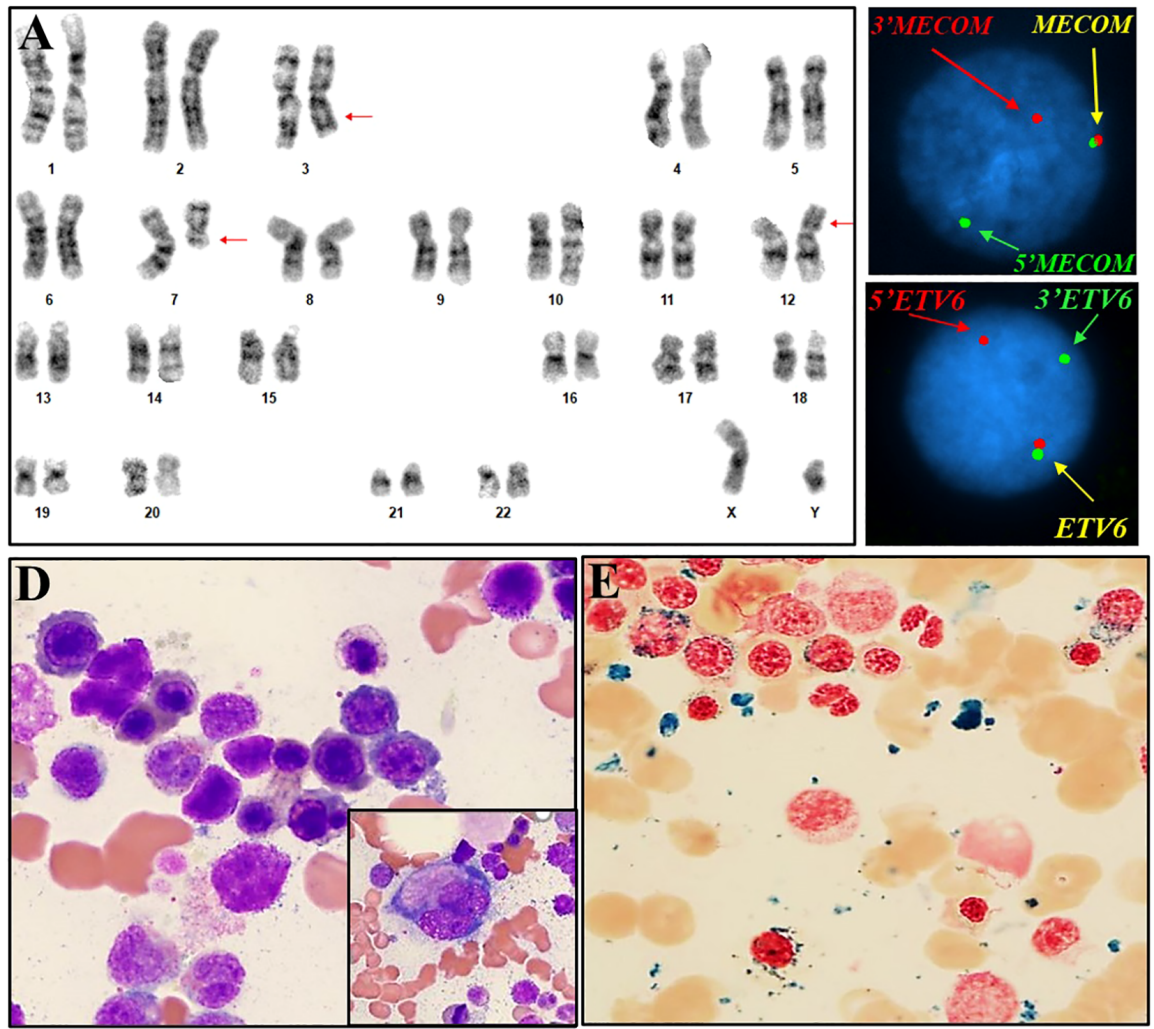
Cytogenetic, FISH and histomorphological findings of case #2. **(A)** Representative karyogram of translocation 46,XY,t(3;12)(q26.2;p13),del(7)(q11.2q32) in 10/20 metaphases. Arrows indicate aberrant chromosomes. **(B)** FISH using dual-color break-apart probes for *MECOM* 3q26.2 demonstrating one normal fusion signal, one split signal for 5’ end region of *MECOM* (green), and one split signal for the 3’ end region of *MECOM* (red), consistent with rearrangement at *MECOM* locus. **(C)** FISH using dual-color break-apart probes for *ETV6* at 12p13.1 demonstrating one normal fusion signal, one split signal for 5’ end region of *ETV6* (red), and one split signal for 3’ end region of *ETV6* (green), consistent with rearrangement at *ETV6* locus. **(D)** Bone marrow aspirate showing dysplasia in erythroid precursors with irregular nuclear contours, budding, basophilic stippling and megaloblastoid features, megakaryocyte with dysplasia and hypolobated morphology (inlet). **(E)** Ringed sideroblasts (iron stain).

Case #2 is a 74-year-old male who presented to the clinic with progressive fatigue and dyspnea. The patient had a history of a long-standing anemia and was diagnosed with a myelodysplastic/myeloproliferative neoplasm (MDS/MPN) with a *JAK-2* mutation at an outside hospital approximately three years prior. He had been closely monitored for 34 months with no remarkable events until he developed progressive anemia, high ferritin and iron saturation, and negative HFE mutations. His platelet count and white blood cell count remained normal. The bone marrow examination revealed a hypercellular bone marrow (90%) with trilineage hematopoiesis, dysplastic erythroid hyperplasia, increased megakaryocytic proliferation with dysplasia, increased ringed sideroblasts (34% of bone marrow erythroid precursors), mild increased blasts (5-7%), and mild diffuse reticulin fibrosis, consistent with the patient’s known history (**Table 1**, **Figures 2D, E**). The patient was started on treatment with azacytidine; the hemoglobin level increased but remained low. By cycle 4, the patient continued to feel fatigued and showed no improvement in anemia. He declined a repeat bone marrow biopsy and was transferred to hospice care. The patient was deceased a month later (**Table 1**).

## 3 Methods

### 3.1 Histopathological examination

Flow cytometric immunophenotyping was performed on cell suspensions prepared from fresh bone marrow specimens using standard methods. A 7-color staining procedure was conducted with the following commercially available monoclonal antibodies: CD2, CD3, CD4, CD5, CD7, CD8, CD10, CD13, CD14, CD16, CD19, CD20, CD22, CD23, CD33, CD34, CD38, CD45, CD56, CD57, CD64, CD71, CD117, HLA-DR, kappa, lambda, IgG1 (FITC), IgG1 (PE), MPO, TdT, cCD3, cCD22, cCD79a, CD11b, CD11c and CD235a (Becton Dickinson Immunocytometry System, Beckman Coulter Inc., Dako Inc.). Bone marrow smears, core biopsies and immunohistochemistry staining were processed and analyzed with standard protocols or following the manufacturer’s instructions for hematological neoplasms (Ventana Medical Systems).

### 3.2 Conventional cytogenetic and interphase fluorescence *in situ* hybridization studies

Chromosome analyses were performed on metaphases harvested from 24- and 48-hour cultured, unstimulated bone marrow cells. Standard GTW-banding procedures were followed. Interphase FISH was conducted using dual color *RPN1/MECOM* translocation, *MECOM* break-apart, and *ETV6* break-apart probe sets (CytoTest Inc., Rockville, MD, USA). Karyotyping and scoring of metaphases as well as FISH images capturing and analysis, were performed with the ASI system (Applied Spectrum Imaging, Carlsbad, CA, USA). The karyograms and FISH signals were described according to the International System for Human Cytogenetic Nomenclature (ISCN) 2020.

### 3.3 Mutation analysis

A next-generation sequencing (NGS)-based analysis was performed to detect somatic mutations in the coding sequences of 73 genes that are recurrently mutated in hematological neoplasms, as described previously (5). The cutoff for common hotspot variants was set at 5%, while for other variants it was 10%. Pathological sequence variants were described according to guidelines by the Human Genome Variation Society (http://www.hgvs.org/mutnomen) based on the genomic reference sequence of genome build hg19.

### 3.4 Literature review and data mining

A literature review was performed by searching published articles in PubMed using keywords “*MECOM*”, “3q”, “t(3;12)” and “acute leukemia”. The search was conducted exclusively in English and included studies published until November 2024. No specific inclusion or exclusion criteria were applied, except for the exclusion of non-English articles. Fusion partner genes of *MECOM* were identified through this literature review, as well as by searching the Atlas of Genetics and Cytogenetics in Oncology and Hematology (https://atlasgeneticsoncology.org) and the Mitelman Database of Chromosome Aberrations and Gene Fusions in Cancer (https://mitelmandatabase.isb-cgc.org). Functional annotation and enrichment analysis were conducted using online bioinformatics tools, including the Gene Ontology (GO) knowledgebase (6, 7) and Enrichr-KG (https://maayanlab.cloud/enrichr-kg) (8). Notably, since most of the selected cases in the literature were diagnosed before the recently published WHO 5th classification of myeloid neoplasms, their original diagnoses and classifications are retained in this report.

## 4 Results

### 4.1 Conventional cytogenetics, fluorescent *in situ* hybridization, and molecular genetics findings

In case #1, a conventional cytogenetics study observed a karyogram of 46,XX,t(3;12)(q26.2;p13.1)[20] (**Figure 1A**). FISH testing with the AML panel probes was negative, except for the presence of three copies of the *MECOM* probes with the *RPN1/MECOM* translocation probe set (data not shown), suggesting a *MECOM* rearrangement with an unknown partner. Reflex testing using gene-specific break-apart probes revealed a signal pattern indicative of rearrangement of the *MECOM* gene at 3q26.2 and the *ETV6* gene at 12p13.1, consistent with a reciprocal translocation between *MECOM* and *ETV6* and the t(3;12) observed in the conventional cytogenetics study (**Figures 1A-C**). NGS-based gene panel analysis revealed pathogenic mutations in genes *EZH2* and *SF3B1* (**Table 1**). In case #2, a conventional cytogenetics study observed a karyogram of 46,XX,t(3;12)(q26.2;p13.1),del (7)(q11.2q32)[10/20] (**Figure 2A**). Rearrangements of the *MECOM* gene and the *ETV6* gene were confirmed by FISH studies (**Figures 2B, C**). NGS-based gene panel analysis revealed pathogenic mutations in the genes *ASXL1, DNMT3A, JAK2, SETBP1* and *SF3B1* (**Table 1**).

### 4.2 Online data mining, pathway, and gene ontology analysis

Myeloid neoplasms with structural alterations involving the chromosomal locus 3q26.2, as reported in the Atlas of Genetics and Cytogenetics in Oncology and Hematology, the Mitelman Database of Chromosome Aberrations and Gene Fusions in Cancer, and various literature reports (2, 9–11) were reviewed.

Thirty-five known genes located on 13 different chromosomes at 30 genomic loci involved in structural rearrangements with the *MECOM* locus at the 3q26.2 were documented, including translocations, inversions and insertions, either balanced or unbalanced (**Table 2**). Clinico-pathological and molecular genetics data are summarized, showing an overall aggressive presentation, poor treatment response and shorter survival time (**Table 2**). Gene ontology analysis, functional annotation, and pathway hunting were further attempted on the two groups of genes with online bioinformatic tools, the Gene Ontology (GO) knowledgebase (6, 7) and Enrichr-KG (https://maayanlab.cloud/enrichr-kg) (8, 12, 13). GO analysis identified “regulation of DNA-templated transcription” (GO: 00006355; https://www.ebi.ac.uk/QuickGO/term/GO:0006355) and “negative regulation of DNA-templated transcription” (GO:0045892; https://www.ebi.ac.uk/QuickGO/term/GO:0045892) as the top annotated biological processes, each comprising 13 and 10 genes, respectively (**Supplementary Table 1**, **Supplementary Figures 1A, B**).

**Table 2:** Structural abnormalities resulting in chimeric gene fusion between *MECOM* locus at 3q26.2 and various partner genomic loci, with either known or unknown gene content in hematologic neoplasms, as documented in the public databases and literature.

Cytogenetic abnormality*	Partner gene	Associated Conditions^&^
t(1;3)(q32; q26.2)	Unknown	AML/MDS
ins(1;3)(p22;q24q26.2)	Unknown	AML
t(2;3)(p14;q26)	Unknown	AML
t(2;3)(p16.1;q26.2)	*BCL11A*	AML
t(2;3)(p21;q26.2)	*THADA*	AML
t(2;3)(p15-23;q26-27)	Unknown	AML, CML-BC, MPN, MDS
del(3)(q25.33q26.2)	*IL12A-AS1*	AML
ins(3;3)(q26.2;q21.3q26.2)	*RPN1, GATA2*	AML, t-AML, s-AML, CML-BC, MDS
inv(3)(p12q26.2)	Unknown	CML
inv(3)(p24.1q26.2)	*TGFBR2*	AML, MDS
inv(3)(q13.33q26.2)	*GTF2E1, STXBP5L*	AML
inv(3)(q21.3q26.2)	*RPN1, GATA2*	AML, t-AML, s-AML, CML-BC, MPN, MDS
inv(3)(q23q26.2)	Unknown	CML
t(3;3)(p24;q26.2)	Unknown	MDS
t(3;3)(q21.3;q26.2)	*RPN1, GATA2*	AML, t-AML, s-AML, CML-BC, MPN, MDS
t(3;3)(q25.33;q26.2)	*SMC4*	AML
t(3;3)(q26.2;q26.31)	*FNDC3B*	AML
t(3;3)(q26.2;q27.3)	*EIF4A2*	AML
t(3;4)(q26;p15.32)	*PROM1, CD38*	AML
t(3;5)(q26.2;q31)	*H2AFY*	AML
t(3;5)(q26.2;q31.1)	*MACROH2A1*	AML
t(3;5)(q26.2;q34)	Unknown	AML
ins(6;3)(q21;q21q26)	*CD164*	AML
t(3;6)(q26;p22.3)	*JARID2*	AML
t(3;6)(q26.2;q25.3)	*ARID1B*	AML
ins(3;7)(q26.2;q34q22)	*TRB*	AML
t(3;7)(q26;p22.1)	*TNRC18, FBXL18*	AML
t(3;7)(q26;q21.12)	*DMTF1*	AML
t(3;7)(q26.2;q21.2)	*CDK6*	AML, CML-BC, MDS
ins(8;3)(q24.1;q26.2q26.2)	Unknown	MDS
t(3;8)(q26.2;p23.1)	*TNKS, MSRA*	AML
t(3;8)(q26.2;q24.1)	*PVT1*	AML, t-AML, MDS, t-MDS
t(3;8)(q26.2;q24.21)	*MYC*	AML
t(3;8)(q26.2;q24.23)	*FAM135B*	AML
t(3;9)(q26.2;p23)	Unknown	T-cell NHL**^#^**
t(3;10)(q26;q21.2)	*ARID5B*	AML
t(3;10)(q26;q22)	Unknown	MDS
t(3;11)(q26.2;p15)	Unknown	CML
t(3;11)(q26;q24.1)	*HSPA8*	AML
ins(12;3)(p13;q21q26.2)	Unknown	MDS
t(3;12)(q26.2;p13.2)	*ETV6*	AML, CML-BC, MDS
t(3;12)(q26.2;q21)	Unknown	t-AML
t(3;16)(q26;q22.1)	*TANGO6, HAS3*	AML
t(3;17)(q26.2;q22)	Unknown	AML, CML-BC, MPN
t(3;18)(q26.2;q11)	Unknown	MDS
t(3;18)(q26;q21.2)	*TCF4*	AML
t(3;21)(q26.2;q11.2)	*NRIP1*	AML, MDS
t(3;21)(q26.2;q22.12)	*RUNX1*	AML, t-AML, CML-BC, MDS, t-MDS
?der(3)(?->3q26.2::3q27.2->)?	*TRA2B*	AML

*Complex alterations with three or more breakpoints are not included.

AML, acute myeloid leukemia; t-AML, therapy related acute myeloid leukemia; s-AML, secondary acute myeloid leukemia; CML-BC, chronic myelogenous leukemia in blast crisis; MPN, myeloproliferative neoplasms; MDS, myelodysplastic syndrome; t-MDS, therapy related myelodysplastic syndrome; NHL, non-Hodgkins lymphoma.

#a single case only.

Given the suggested role in transcription for genes involved in the *MECOM* rearrangement, Enrichr-KG was utilized to search for transcription binding sites and the corresponding factors bound to those sites enriched from ChEA2022 libraries across the aforementioned 35 genes. Chromatin immunoprecipitation sequencing (ChIP-seq) studies on a human leukemia cell line SEM documented from ChEA2022 study (14), identified 18 of the 35 genes as putative targets of the transcription elongation factor AF4 (**Supplementary Table 2**, **Supplementary Figure 2**).

Furthermore, although NGS-based cancer mutational panel testing data were available in literature for only a limited number of cases with *MECOM* rearrangement, 38 genes carrying pathogenic mutations co-existing with *MECOM* rearrangement were identified, including those in the current two cases (**Supplementary Table 3**). Enrichr-KG gene set enrichment analysis of these 38 genes showed that 20 of them are also targets of AF4 in SEM cells based on the ChIP-seq study (14) (**Supplementary Figure 3**).

## 5 Discussion

*MECOM*/3q26 abnormalities result in overexpression of MECOM rather than generating a chimeric gene. These abnormalities mainly involve the juxtaposition of regulatory sequences near *MECOM*, functioning as a super enhancer or a promoter (15). In myeloid neoplasms, t(3;12)(q26.2;p13.1) resulting in an apparent *MECOM::ETV6* fusion was found in less than 0.1% of these cases. The corresponding neoplastic transformation is believed to be triggered by MECOM overexpression driven by the *ETV6* promoter (4, 10, 15). To date, only 78 cases have been reported in literature. Through routine conventional cytogenetics study, we have identified two new cases with t(3;12).

In case #1, t(3;12) was found at the diagnosis, indicative of a driver event, while in case #2, it appeared as an acquired event during disease progression. Deletion 7q was observed in case #2, consistent with -7/7q being the most common secondary chromosomal abnormality in patients with *MECOM* rearrangements (Tang et al., 2019a, Tang et al., 2019b). Reports also suggest that t(3;12) is associated with the transformation of MDS to AML (2, 16), which was found in case #1. However, in contrast to the significantly high failure rates of therapy in literature (2, 16) and along with a quick AML transformation, case #1 is doing exceptionally well at the latest follow-up more than two years post-HSCT, suggesting that aggressive induction and consolidation therapy along with transplant are an effective approach treating patients with t(3;12). The poor prognosis of case #2 is most likely due to the presence of an additional 7q deletion, which by itself is a high-risk genetic marker in myeloid neoplasms. Despite the poor prognosis of case #2, his blast count was only 5-7%, which is consistent with findings that the prognosis of MDS or AML cases with *MECOM* rearrangements is not dependent on blast count (11). Although the expression level of MECOM was not directly measured, it is hypothesized to be elevated in both cases. Intriguingly, a recent study demonstrated that the hypomethylating agent 5-aza-2-deoxycytidine can induce widespread apoptosis in MECOM-high leukemia cells, highlighting its potential as a therapeutic agent for leukemia with MECOM overexpression (17). However, in case #1, the patient was initially treated with decitabine, a hypomethylating agent, upon diagnosis of MDS-EB2, but still progressed to AML. In case #2, the patient was treated with Azacitidine, a similar hypomethylating agent, but did not respond well, either. Although interesting, given that the current report includes only two cases, the clinical outcomes observed cannot be considered statistically conclusive. Both cases showed dysplasia affecting at least two lineages, consistent with dysplasia being a distinct morphological feature associated with *MECOM* rearrangements (2, 16).

To date, more than 30 genomic loci have been reported to fuse with the *MECOM* locus in human myeloid neoplasms. This diversity raises the question of whether any common traits are shared among these the fusion partners? Specifically, are there links among different *MECOM* rearrangement partners? Through a review of the literature and online database searches, 35 known genes at 30 genomic loci located at the fusion breakpoints with *MECOM* were identified. We demonstrate here that AF4 target genes are overrepresented among these fusion partners.

AF4 is primarily known to be the fusion partner of the *KMT2A/MLL* gene in infant acute lymphoblastic leukemia with chromosomal translocation t(5;12)(q31;q23). The *AF4* gene (also known as ALF transcription elongation factor 4 or *AFF4*) is located on 5q31.1, and encodes a protein that belongs to the AF4/LAF4/FMR2 (ALF) family. AF4 directly interacts with ELL proteins (ELL, ELL2 or ELL3) and the P-TEFb complex to form the super elongation complex (SEC). SEC then stimulates transcript elongation by converting the RNA polymerase II (Pol II) into its elongating form with an enhanced catalytic rate at multiple sites along the DNA. Additionally, the AF4 family/ENL family/P-TEFb complex (AEP) is capable of activating transcription by binding to acetylated H3K9/18/27. Further functional verification of AF4 involvement in *MECOM* rearrangement-based myeloid neoplasms will help elucidate the corresponding molecular etiology and identify therapeutic targets (18–21).

The limitations of this study include:

1. Reported data in literature primarily focus on clinical observations. We analyzed and predicted biological functions using multiple database searches and online bioinformatics tools. To advance *in vivo* proof-of-concept investigations, initial steps could include isolating endogenously occurring *MECOM::ETV6* fusion products from the primary leukemia patient cells, mapping the nucleotide sequences of the breakpoints, characterizing potential functional elements, and generating immortalized cell lines using the chimeric fusion genes.
2. There is a lack of genome-wide analyses of *MECOM-*rearranged leukemia. In fact, very limited data is available in literature. Only recently has NGS-based cancer panel sequencing begun to be tested on these patients, and the data is not available for the majority of reported cases. Given the complex biological nature of human cancer, genome-wide analyses, including epigenomics, genomics, proteomics and metabolomics studies, are warranted to further investigate and characterize key signaling pathways and networks and critical elements associated with leukemia containing the *MECOM* rearrangement.
3. Due to limited resources, we were unable to but propose to conduct the following studies: isolation of the full-length *MECOM::ETV6* fusion product, identification of the breakpoints, characterization of the functional domains of both *MECOM* and *ETV6* included in the chimeric gene, and *in vivo* functional studies accessing MECOM expression and the responses of predicted signaling pathways. Additionally, future *in vitro* and *in vivo* studies are needed to provide experimental evidence evaluating the functional role of AF4 in MECOM-mediated leukemogenesis.
4. This study is based on only two cases, which limits the general applicability of the conclusions. Future research with larger sample sizes and potential collaborations is warranted to enhance the robustness and applicability of the findings.
5. The potential complex interplay between co-existing genetic mutations and *MECOM* rearrangements in disease progression, clonal evolution, and treatment response requires further exploration. Molecular classification of these mutations could guide the selection of targeted therapies, such as inhibitors of specific pathways. By incorporating clinical data, such as electronic medical records, the impact of these mutations on the efficacy of standard treatments like chemotherapy or stem cell transplantation can be further studied. Due to the limited availability of such data for the cases studied in this manuscript, the genetic analyses currently require further refinement.

Several questions remain to be answered. For example, is t(3;12) sufficient to transform myeloid progenitors? Is AF4 a potential link between *MECOM*-rearranged leukemia and CTCF pathways, given that both bind to acetylated H3K9/18/27? Is there any molecular mechanistic overlap between *MECOM* and *KMT2A* rearranged leukemia? Since *AF4* is one of the partner genes for *KMT2A*, could therapeutic approaches currently utilized or studied for leukemia with *KMT2* rearrangement be applicable to those with *MECOM* rearrangement?

In summary, we have described the clinical, histomorphological and genetic features of two new patients with myeloid neoplasms carrying a t(3;12). We demonstrate that AF4 is likely an upstream regulator in physiologic MECOM-dependent transcriptional pathways, and propose that it might be one of the major mechanisms of MECOM-mediated leukemic transformation. Our study provides further insight into the molecular etiology of *MECOM* translocation-based myeloid neoplasms. We propose that the *AF4* gene and the transcription elongation control pathway could be potential targets for the treatment of *MECOM*-associated myeloid neoplasms. Additionally, our results underscore the significance of conducting routine conventional cytogenetics assessment in myeloid neoplasms. For future clinical and research studies of acute myeloid leukemia with t(3;12), we suggest grouping all patients with various chromosomal rearrangements involving the *MECOM* locus at 3q26.2 to address the issue of small sample sizes.

## Data Availability

The original contributions presented in the study are included in the article/**Supplementary Material**, further inquiries can be directed to the corresponding author/s.

## References

[1] Hinai AA Valk PJ. Review: Aberrant EVI1 expression in acute myeloid leukaemia. Br J Haematol. (2016) 172:870–8. doi: 10.1111/bjh.2016.172.issue-6 26729571

[2] Tang Z Tang G Hu S Patel KP Yin CC Wang W. Deciphering the complexities of MECOM rearrangement-driven chromosomal aberrations. Cancer Genet. (2019) 233-234:21–31. doi: 10.1016/j.cancergen.2019.03.002 31109591

[3] Lugthart S Groschel S Beverloo HB Kayser S Valk PJ van-Zelderen-Bhola SL. Clinical, molecular, and prognostic significance of WHO type inv(3)(q21q26.2)/t(3;3)(q21;q26.2) and various other 3q abnormalities in acute myeloid leukemia. J Clin Oncol. (2010) 28:3890–8. doi: 10.1200/JCO.2010.29.2771 20660833

[4] Ronaghy A Hu S Tang Z Wang W Tang G Loghavi S. Myeloid neoplasms associated with t(3;12)(q26.2;p13) are clinically aggressive, show myelodysplasia, and frequently harbor chromosome 7 abnormalities. Mod Pathol. (2021) 34:300–13. doi: 10.1038/s41379-020-00663-z 33110238

[5] Simen BB Yin L Goswami CP Davis KO Bajaj R Gong JZ. Validation of a next-generation-sequencing cancer panel for use in the clinical laboratory. Arch Pathol Lab Med. (2015) 139:508–17. doi: 10.5858/arpa.2013-0710-OA 25356985

[6] Ashburner M Ball CA Blake JA Botstein D Butler H Cherry JM. Gene ontology: tool for the unification of biology. Gene Ontol Consortium Nat Genet. (2000) 25:25–9. doi: 10.1038/75556 PMC3037419 10802651

[7] Gene Ontology C Aleksander SA Balhoff J Carbon S Cherry JM Drabkin HJ. The gene ontology knowledgebase in 2023. Genetics. (2023) 224:14. doi: 10.1093/genetics/iyad031 PMC10158837 36866529

[8] Evangelista JE Xie Z Marino GB Nguyen N Clarke DJB Ma’ayan A. Enrichr-KG: bridging enrichment analysis across multiple libraries. Nucleic Acids Res. (2023) 51:W168–W79. doi: 10.1093/nar/gkad393 PMC10320098 37166973

[9] Gao J Gurbuxani S Zak T Kocherginsky M Ji P Wehbe F. Comparison of myeloid neoplasms with nonclassic 3q26.2/MECOM versus classic inv(3)/t(3;3) rearrangements reveals diverse clinicopathologic features, genetic profiles, and molecular mechanisms of MECOM activation. Genes Chromosomes Cancer. (2022) 61:71–80. doi: 10.1002/gcc.23004 34668265

[10] Ottema S Mulet-Lazaro R Beverloo HB Erpelinck C van Herk S van der Helm R. Atypical 3q26/MECOM rearrangements genocopy inv(3)/t(3;3) in acute myeloid leukemia. Blood. (2020) 136:224–34. doi: 10.1182/blood.2019003701 32219447

[11] Summerer I Haferlach C Meggendorfer M Kern W Haferlach T Stengel A. Prognosis of MECOM (EVI1)-rearranged MDS and AML patients rather depends on accompanying molecular mutations than on blast count. Leuk Lymphoma. (2020) 61:1756–9. doi: 10.1080/10428194.2020.1737689 32189545

[12] Lachmann A Xu H Krishnan J Berger SI Mazloom AR Ma’ayan A. ChEA: transcription factor regulation inferred from integrating genome-wide ChIP-X experiments. Bioinformatics. (2010) 26:2438–44. doi: 10.1093/bioinformatics/btq466 PMC2944209 20709693

[13] Keenan AB Torre D Lachmann A Leong AK Wojciechowicz ML Utti V. ChEA3: transcription factor enrichment analysis by orthogonal omics integration. Nucleic Acids Res. (2019) 47:W212–W24. doi: 10.1093/nar/gkz446 PMC6602523 31114921

[14] Benito JM Godfrey L Kojima K Hogdal L Wunderlich M Geng H. MLL-rearranged acute lymphoblastic leukemias activate BCL-2 through H3K79 methylation and are sensitive to the BCL-2-specific antagonist ABT-199. Cell Rep. (2015) 13:2715–27. doi: 10.1016/j.celrep.2015.12.003 PMC4700051 26711339

[15] Li Q Wang F Zhang X Liu S Sun MZ Yan J. The ETV6-MECOM fusion protein promotes EMT-related properties by repressing the transactivation activity of E-cadherin promoter in K562 leukemia cells. Biochem Biophys Rep. (2024) 38:101667. doi: 10.1016/j.bbrep.2024.101667 38405662 PMC10884757

[16] Tang G Hu S Wang SA Xie W Lin P Xu J. t(3;8)(q26.2;q24) often leads to MECOM/MYC rearrangement and is commonly associated with therapy-related myeloid neoplasms and/or disease progression. J Mol Diagn. (2019) 21:343–51. doi: 10.1016/j.jmoldx.2018.10.005 30576868

[17] Li F He W Geng R Xie X. Myeloid leukemia with high EVI1 expression is sensitive to 5-aza-2’-deoxycytidine by targeting miR-9. Clin Transl Oncol. (2020) 22:137–43. doi: 10.1007/s12094-019-02121-y 31054042

[18] Lin C Smith ER Takahashi H Lai KC Martin-Brown S Florens L. AFF4, a component of the ELL/P-TEFb elongation complex and a shared subunit of MLL chimeras, can link transcription elongation to leukemia. Mol Cell. (2010) 37:429–37. doi: 10.1016/j.molcel.2010.01.026 PMC2872029 20159561

[19] Yokoyama A Lin M Naresh A Kitabayashi I Cleary ML. A higher-order complex containing AF4 and ENL family proteins with P-TEFb facilitates oncogenic and physiologic MLL-dependent transcription. Cancer Cell. (2010) 17:198–212. doi: 10.1016/j.ccr.2009.12.040 20153263 PMC2824033

[20] Yokoyama A. Leukemogenesis via aberrant self-renewal by the MLL/AEP-mediated transcriptional activation system. Cancer Sci. (2021) 112:3935–44. doi: 10.1111/cas.v112.10 PMC8486200 34251718

[21] Che Z Liu X Dai Q Fang K Guo C Yue J. Distinct roles of two SEC scaffold proteins, AFF1 and AFF4, in regulating RNA polymerase II transcription elongation. J Mol Cell Biol. (2024) 15:13. doi: 10.1093/jmcb/mjad049 PMC11113081 37528066

