# Supplementary material for: Case Report: Myeloid neoplasms with the t(3;12)(q26.2;p13.1)/MECOM-ETV6 translocation: report of two new cases and review of the literature
Source: Front Oncol. 2025 Apr 4;15:1526044. doi: 10.3389/fonc.2025.1526044 (PMC12006008; doi:10.3389/fonc.2025.1526044)
Supplement: Supplementary file 1 [file DataSheet1.docx]

**Supplementary material**

**Supplementary Table 1.** The top two annotated biological process, “regulation of DNA-templated transcription” and “negative regulation of DNA-templated transcription”, identified in GO database.

| **GO:0006355** | **GO:0045892** |
| --- | --- |
| *ARID5B* | *ARID5B* |
| *GATA2* | *JARID2* |
| *CD38* | *RUNX1* |
| *ARID1B* | *HSPA8* |
| *JARID2* | *ETV6* |
| *MYC* | *MYC* |
| *RUNX1* | *NRIP1* |
| *HAS3* | *CD38* |
| *TCF4* | *BCL11A* |
| *BCL11A* | *MACROH2A1* |
| *ETV6* |  |
| *DMTF1* |  |
| *HSPA8* |  |

**Supplementary Table 2.** Chromatin immunoprecipitation sequencing (ChIP-seq) studies on a human acute lymphoblastic leukemia cell line SEM documented from ChEA2022 libraries showing 18 of the 35 genes were putative targets of the transcription elongation factor AF4.

| **AF4 26711339** |
| --- |
| *BCL11A* |
| *ARID5B* |
| *MSRA* |
| *TGFBR2* |
| *ARID1B* |
| *TRA2B* |
| *CD164* |
| *EIF4A2* |
| *HSPA8* |
| *FBXL18* |
| *DMTF1* |
| *CDK6* |
| *SMC4* |
| *ETV6* |
| *JARID2* |
| *RUNX1* |
| *MYC* |
| *PROM1* |

**Supplementary Table 3**. 38 genes known to be mutated in *MECOM*-rearranged cases by NGS-based cancer mutational panel testing.

| *ARID1A* |
| --- |
| *ASXL1* |
| *BCOR* |
| *BRCA2* |
| *CALR* |
| *CBL* |
| *CCND2* |
| *CEBPA* |
| *CUX1* |
| *DDX41* |
| *DNMT3A* |
| *ETV6* |
| *EZH2* |
| *FLT3* |
| *GATA2* |
| *IDH1* |
| *IDH2* |
| *IKZF1* |
| *JAK2* |
| *JAK3* |
| *KIT* |
| *KRAS* |
| *MPL* |
| *NF1* |
| *NRAS* |
| *PHF6* |
| *PTPN11* |
| *RAD21* |
| *RUNX1* |
| *SETBP1* |
| *SF3B1* |
| *SRSF2* |
| *STAG2* |
| *TET2* |
| *TP53* |
| *U2AF1* |
| *WT1* |
| *ZRSR2* |

**
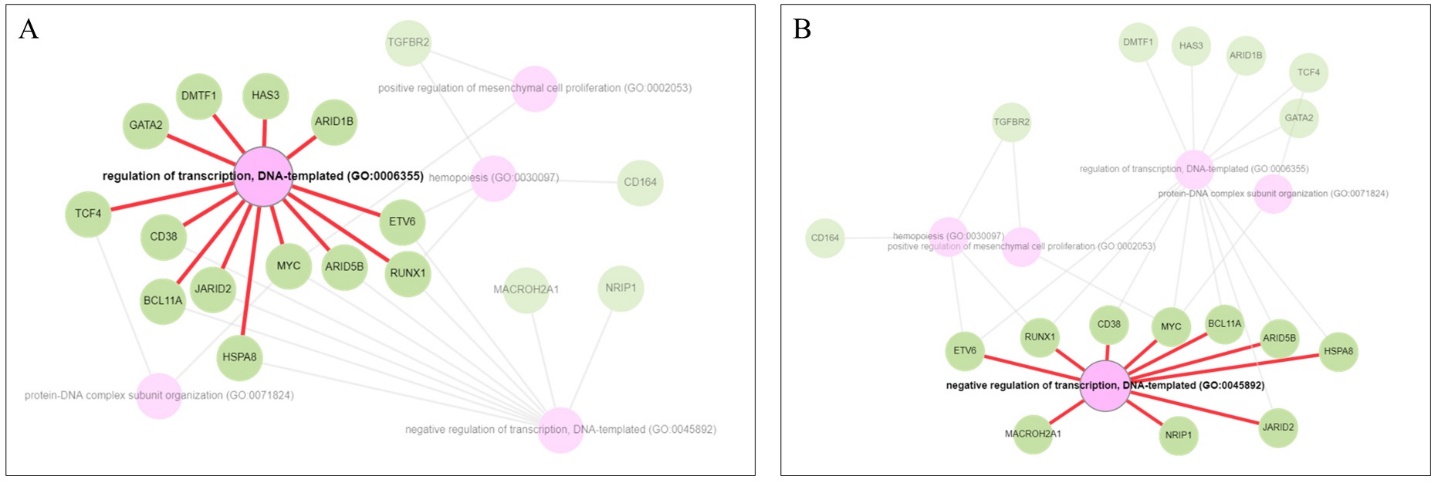
**

**Supplementary Figure 1**. Gene Ontology biological process analysis of the 35 genes located in the vicinity of partner loci of *MECOM*. **(A)** 13 genes belong to the biological process GO:0006355 regulation of DNA-templated transcription, including *ARID5B, GATA2, CD38, ARID1B, JARID2, MYC, RUNX1, HAS3, TCF4, BCL11A, ETV6, DMTF1*, and *HSPA8*. **(B)** 10 genes belong to the biological process GO:0045892 negative regulation of DNA-templated transcription, including *ARID5B, JARID2, RUNX1, HSPA8, ETV6, MYC, NRIP1, CD38, BCL11A,* and *MACROH2A1*. The Gene Ontology (GO) knowledgebase is the world’s largest source of gene functional information.

**
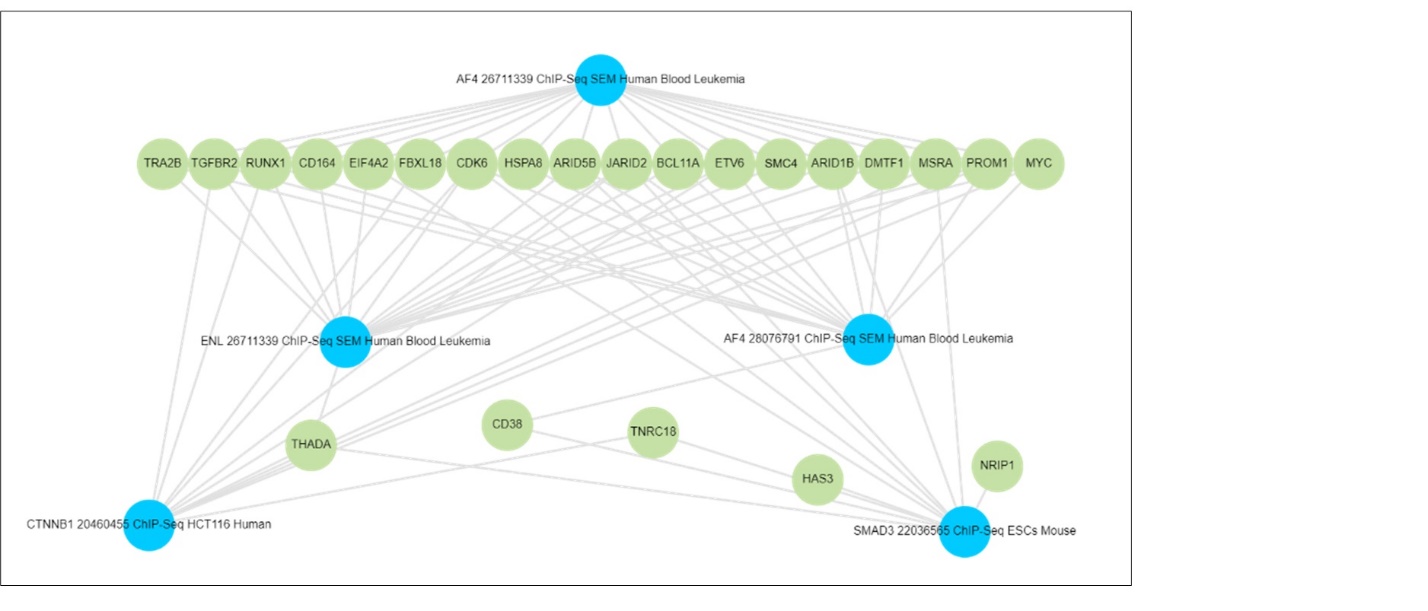
**

**Supplementary Figure 2**. Enrichr-KG gene set enrichment analysis of the 35 genes located in the vicinity of partner loci of *MECOM*, showing 18 of them are putative targets of the transcription factor AF4. The Enrichr-KG performs is a web-based search engine identifying enriched gene set from published data libraries from various types of genome-wide researches in transcription, pathways, ontologies, diseases/drugs, cell types, etc. ChEA2022 transcription factor binding site profiles 2022, is one of the Enrichr-KG curated libraires, containing putative target genes curated from published ChIP-chip, ChIP-seq, and other transcription factor binding site profiling studies.


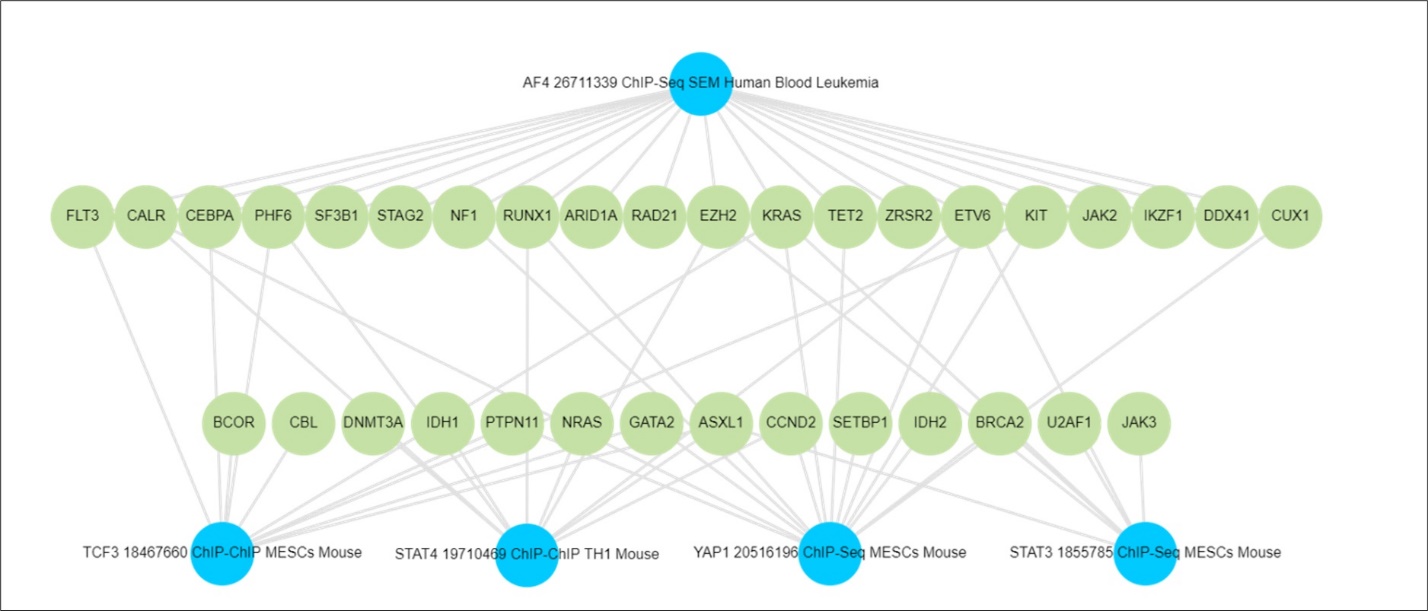


**Supplementary Figure 3.** Enrichr-KG gene set enrichment analysis of the 38 genes mutated in myeloid neoplasm cases with *MECOM* rearrangements, showing 20 of them are putative targets of the transcription factor AF4. ChEA2022 transcription factor binding site profiles 2022, is one of the Enrichr-KG curated libraires, containing putative target genes curated from published ChIP-chip, ChIP-seq, and other transcription factor binding site profiling.
